# Prevalence, Patterns, and Determinants of Malaria and Malnutrition in Douala, Cameroon: A Cross-Sectional Community-Based Study

**DOI:** 10.1155/2021/5553344

**Published:** 2021-07-12

**Authors:** Loick Pradel Kojom Foko, Nicolas Policarpe Nolla, Hervé Nyabeyeu Nyabeyeu, Calvin Tonga, Leopold Gustave Lehman

**Affiliations:** ^1^Department of Animal Biology, Faculty of Science, The University of Douala, P.O. Box 24157, Douala, Cameroon; ^2^Department of Biochemistry, Faculty of Science, The University of Douala, P.O. Box 24157, Cameroon; ^3^Department of Biological Sciences, Faculty of Medicine and Pharmaceutical Sciences, The University of Douala, P.O. Box 2701, Douala, Cameroon

## Abstract

**Background:**

Malaria and malnutrition are major public health problems in developing countries. This studywas mainly focused on the prevalence, patterns, and predictors of these conditions and their associations.

**Methods:**

A cross-sectional community study was conducted from February to March 2018 among 281 participants living in two districts in Douala. A questionnaire was used to collect sociodemographic information and parasitological and anthropometric data of participants. Nutritional status was determined using age, weight, and height. Body mass index for age (BMIAZ), height-for-age (HAZ), weight-for-age (WAZ), and weight-for-height (WHZ) *Z* scores were computed based on the World Health Organization growth reference curves. Malaria infection was diagnosed using light-emitting diode fluorescence microscopy.

**Results:**

The overall prevalence of malaria was 18.9%, mostly asymptomatic cases. Malaria infection was associated with study site (*p* = 0.04), age (*p* = 0.01), WAZ (*p* = 0.0049), HAZ (*p* = 0.03), and BMI (*p* = 0.02). The overall prevalence of malnutrition was 43.1%, and stunting was the main form of malnutrition recorded in children under five years of age (23.6%). The risk of being stunted in this group was about quintupled in malaria-infected participants (ARR = 4.70; *p* = 0.02). In those aged 5-19 years, the prevalence of underweight was significantly higher in malaria-positive individuals as compared to their negative counterparts (*p* = 0.02). The overall prevalence of malaria and malnutrition cooccurrence was 8.5% and varied with age (*p* < 0.0001) and study site (*p* = 0.04). *Conclusion and Recommendation*. Malaria was associated with malnutrition among the study participants. Early detection and treatment of these ailments would reduce morbidity and mortality.

## 1. Introduction

Malaria and malnutrition are both important public health problems worldwide, especially in developing countries. The African continent is seriously burdened with malaria and malnutrition as it accounts for the bulk of victims. In 2019, the World Health Organization (WHO) reported that malaria was responsible for 229 million disease cases and 409,000 deaths worldwide; sub-Saharan Africa (sSA) concentrated more than 90% of these statistics [1].

Malnutrition is a global public health problem that mostly affects communities of developing countries under its various forms, including undernutrition (wasting, stunting, and underweight), inadequate vitamins or minerals intake, overweight, obesity, and resulting diet-related noncommunicable diseases [2]. Undernutrition is an underlying cause of almost half (45%) of deaths in children under five worldwide; it increases the susceptibility to infection and exposes to poor physical and cognitive development [3]. In 2018, about 49 million children under 5 years of age were affected by wasting, 149 million by stunting, and 40 million are overweight [4]. Concerning adults, 1.9 billion were overweight or obese while 462 million were underweight [2].

Malaria and malnutrition are causes of concern in Cameroon. Malaria is the main cause of hospital morbidity and mortality, accounting for 40-50% of hospital consultations and 23% of hospitalizations [5]. As regards malnutrition, the prevalence of wasting and stunting is more than 30% in six out of 10 regions of the country. Overall, more than 31% of children less than five years are wasted while this rate peaks at 40% in the Far North region [6].

The relationship between both health problems is still elusive as findings are diverse and conflicting [7, 8]. A better understanding of the relation is of utmost public health importance. Thus, there is a need for further studies on the topic, especially in Cameroon where little data are available [9, 10].

## 2. Aims and Objectives

The aim of this study was to determine the prevalence, patterns, and factors associated with malnutrition and malaria among individuals living in Douala, Cameroon. In addition, the eventual associations between both diseases were also studied.

## 3. Material and Methods

### 3.1. Study Sites

**Figure 1:**
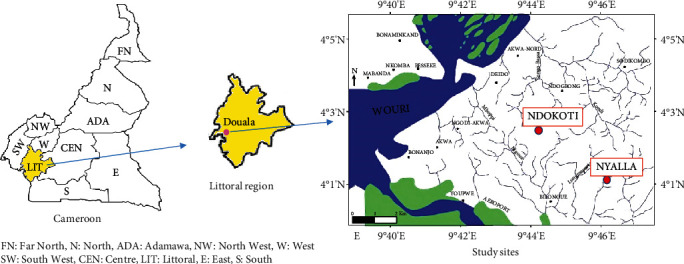
Map of Cameroon depicting the ten regions, Littoral region, and the study sites.

The study was carried out in two districts in the city of Douala (Littoral Region, Cameroon), namely Ndokoti and Nyalla (Figure 1). These two areas are characterized by a topography consisting of mostly flat terrain which encourages close-range construction with inadequate drainage systems as well as the practicing of agriculture. Populations of these districts are highly heterogeneous, but with a predominance of three ethnic groups (*Bamileke*, *Bassa*, and *Duala*). Ndokoti and Nyalla have tropical climate with two seasons, one dry season (October to May) and one rainy season (June to October). The humidity rate is high, with an average temperature ranging from 25°C to 30°C [11–13]. All these characteristics make Ndokoti and Nyalla, two areas, propitious to development of mosquito vectors for malaria transmission. In addition, no studies on malaria and malnutrition were conducted in these districts. These two reasons mainly guided the choice of these districts as study sites.

### 3.2. Study Population and Sampling Size

The study population consisted of children, adolescents, and adults living in the town of Douala. They were enrolled using random sampling method. Sample size was calculated using the Lorentz's formula, as follows: *N* = *p* (1 − *p*) *z*^2^/*d*^2^ where *N* is the sample size, *p* is the prevalence of malaria and malnutrition cooccurrence (8.17%, based on previous findings in the Adamawa Region, Cameroon) [9], *z* is the decision variable at confidence of 95% (*z* = 1.96), and *d* is the sampling-related error risk (*d* = 0.05). The minimum sample size for the study was estimated at 115 participants.

### 3.3. Study Design

A cross-sectional community-based study was carried out from February to March 2018. The objectives and benefits of the study were explained to each participant (i.e., older children,adolescents, and adults), and parents/guardians of children and adolescents. After obtaining written consent from parents/guardians and adult participants along with assent from older children and adolescents, anthropometric and clinical data were collected from each participant. Moreover, a few drops of blood were collected for malaria diagnosis. Finally, participants and their parents/guardians were educated on malaria and control measures.

### 3.4. Participants' Data Collection

Data collection was done through 15-25-minute interviews of participants or their guardians and recording in a survey form.

### 3.5. Clinical Parameters

Axillary body temperature of each participant was measured using an electronic thermometer (Beurer GmbH, Germany). Fever was defined as axillary temperature of >37.5°C [11].

### 3.6. Anthropometric Parameters

Anthropometric measurements of each participant were collected by two trained field workers using standard methods. Height (cm) was measured to the nearest 1 cm with a stadiometer in the recumbent position and erect position for participants aged less than 24 months and those above 24 months, respectively [14]. Body weight (kg) was measured using electronic weighing scale (SANITAS, Hans Dinslage GmbH, Germany) calibrated to the nearest 0.5 kg. Height (H) and body weight (BW) were used for computing body mass index (BMI) using Quetelet's formula as follows: BMI (kg/m^2^) = BW/H^2^.

### 3.7. Assessment of Nutritional Status

Anthropometric indicators including BMI and *Z* scores were used to determine the nutritional status of each participant. Body mass index was used for adolescents and adults while *Z* scores were used for children. Malnutrition among adults was defined as BMI < 18.5 kg/m^2^ (undernutrition), BMI ranging 25-29.9 kg/m^2^ (overweight), and BMI ≥ 30 kg/m^2^ (obesity). BMI ranging between 18.5 and 24.9 kg/m^2^ was considered normal [10, 15]. Weight-for-height *Z* score (WHZ), height-for-age *Z* score (HAZ), and weight-for-age *Z* score (WAZ) less than -2 were used to define wasting, stunting, and underweight, respectively, among those under five years old, while BMI-for-age *Z* (BMIAZ) and HAZ scores were used for those aged 5-19 years. Scores < −3 were indicative of a severe nutritional conditions [10, 15]. The *Z* scores were computed using the WHO Anthro version 3.2.2 (WHO, Geneva, Switzerland) and WHO AnthroPlus version 1.0.4 (WHO, Geneva, Switzerland) software. Children and adolescents were diagnosed as malnourished when at least one of these *Z* scores was less than -2 [10, 15].

### 3.8. Malaria Diagnosis

**Figure 2:**
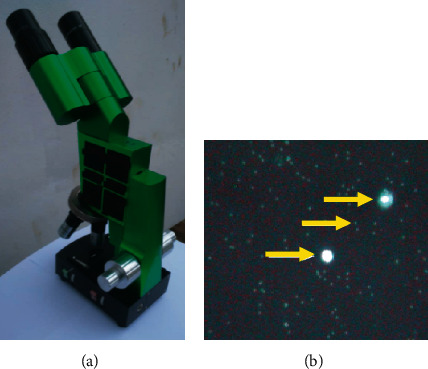
(a) CyScope® microscope (Partec-Sysmex, Japan). (b) Positive malaria slide under observation with CyScope®. Bigger and smaller spots correspond to white blood cells and malaria parasites, respectively (photographs are provided by the authors).

Diagnosis of malaria was performed using light-emitting diode (LED) fluorescence microscopy (FM) (Figure 2). This technique was preferred to light microscopy and rapid diagnostic tests given its interesting operational characteristics (malaria diagnosis < 5 minutes, good labour-effectiveness, microscopic identification of parasites, and battery-operated microscope) and diagnostic performances similar to molecular methods [16–18]. Based on these characteristics, the utilisation of FM allows to overcome limitations of light microscopy and rapid diagnostic tests, especially in community-based studies [16, 19]. Briefly, few drops of blood were collected from finger prick and deposited on slides stained with 4′,6-diamidino-2-phenylindole (DAPI). The preparation was then covered with a cover slip and kept in the dark for 1 minute [20]. Slides were read by skilled microscopists. The results were given to participants or their parents/guardians.

Symptomatic malaria was defined as the presence of malaria parasites using fluorescence microscopy (FM) associated with fever [11]. Asymptomatic malaria was defined as the presence of malaria parasites using FM associated with the absence of fever [11].

### 3.9. Ethical Considerations

The study was approved by the institutional review board (IRB) of the University of Douala (No. CEI-UD/270/09/2015/T). Administrative clearance was sought from Delegation of Public Health for Littoral Region (No. 2596/AS/MINSANTE/DRSPL/BCASS). Written consent forms were obtained from each participant or their parents/guardians before their inclusion in the study. Objectives, gains, and risks of the study were explained to each participants, parents, and guardians in the French or English language. They were informed about the confidential and voluntary aspect of the study and were free to withdraw at any time. The right of children and adolescents not to participate in the study despite parental approval was respected. Positive malaria cases were treated as per national treatment guidelines with artesunate + amodiaquine [21].

### 3.10. Statistical Analyses

All data were verified for consistency, coded, and keyed into an Excel spreadsheet (Microsoft Office 2016, USA) and thereafter analysed with the Statistical Package for Social Science (SPSS) v22 for Windows (SPSS Inc., Chicago, IL, USA) and GraphPad v5.03 for Windows (GraphPad PRISM, Inc., San Diego, CA, USA). Data were presented as percentage (confidence interval at 95%) or mean ± standard deviation (SD) where appropriate. Participants were categorized into three age groups (<5, 5-19, and ≥20 years old) as per the WHO reports on growth indicators assessments [22]. Normality of continuous variables was tested using the Shapiro-Wilk test. The confidence intervals at 95% were computed as described elsewhere [23]. Independent chi-square (*χ*^2^) and Fisher's exact tests were used to compare proportions upon respect of Cochran's rule. Parametric statistical tests including one-way analysis of variance (ANOVA) and unpaired sample Student's tests were used to compare mean values between groups. Their nonparametric versions (Kruskal-Wallis and Mann-Whitney) were used if data failed to respect hypotheses for using these parametric tests. Univariate and multivariate logistic regression models were used to quantify the influence of malaria on nutritional status and conversely. Multivariate logistic regression was used to identify factors associated with the risk of being both malaria infected and malnourished. The resulting odds ratio (OR) were then converted into risk ratio (RR) as described by Zhang and Yu [24]. A *p* value < 0.05 was considered statistically significant.

## 4. Results

### 4.1. Baseline Data of the Participants

Table 1 shows the distribution of the study population with regard to gender and age. Two hundred and eighty-one (281) individuals were included in the study. In total, 62.6% and 37.4% of individuals were females and males, respectively. The age of participants ranged from 1 to 85 years with mean ± SD of 22 ± 19 years.

### 4.2. Prevalence of Malaria Infection

In total, 53 individuals were infected with malaria parasites, thus an overall prevalence of 18.9% (53/281; 95% CI: 14.7-23.8%). Malaria infection rate was significantly lower in the Nyalla district (14.3%, _A_RR = 0.59; 95% CI: 0.33-0.99; *p* = 0.04) and among participants above 20 years (13.8%, _A_RR = 0.48; 95% CI: 0.17-0.97; *p* = 0.01). On the other hand, the malaria prevalence was higher in malnourished individuals as compared to normal individuals (19.8% vs. 18.1%). The risk of malaria infection was higher in malnourished participants, but no association was found (_A_RR = 1.68; 95% CI: 0.95-2.63; *p* = 0.07) (Table 2). Participants infected with malaria parasites were significantly younger than their uninfected counterparts (16.7 ± 16.4 years versus 23.5 ± 19.7 years; *p* = 0.01). Besides, most malaria infection cases were asymptomatic (84.9%, 45/53; 95% CI: 73.0-92.2%).

Using univariate logistic regression model, a statistically significant association was found between malaria infection and WAZ (*p* = 0.0049), HAZ (*p* = 0.03), and BMI (*p* = 0.02). In fact, the risk of malaria infection is reduced by 41% (_A_RR = 0.59; 95% CI: 0.41-0.85), 20% (_A_RR = 0.80; 95% CI: 0.64-0.99), and 6% (_A_RR = 0.94; 95% CI: 0.89-0.99), respectively, when these anthropometric indices increase by one unity. No association was found with WHZ (_A_RR = 0.94; 95% CI: 0.69-1.26; *p* = 0.66) and BMIAS (_A_RR = 0.99; 95% CI: 0.83-1.19; *p* = 0.95).

### 4.3. Prevalence and Factors Associated with Malnutrition

The overall prevalence of malnutrition was 43.1% (121/281; 95% CI: 37.4-48.9%) among participants. The risk of malnutrition was threefold higher (_A_RR = 2.70; 95% CI: 2.08-3.07; *p* < 0.0001) in those aged ≥ 20 years as compared to those less than 5 years (Table 3). In addition, participants suffering from malnutrition were significantly older than their younger counterparts (33.9 ± 21.9 years vs. 13.3 ± 10.2 years; *p* < 0.0001).

### 4.4. Proportion of Different Types of Malnutrition by Age Groups and Gender

The overall proportion of undernutrition was 29.4% (10/34; 95% CI: 16.8-46.2%) among children under five as presented in Table 4, mainly stunting (23.6%, 8/10), with two severe cases. The proportion of participants with stunting was slightly higher in female compared to males (29.4% vs. 17.6%) (Table 4).

Likewise, stunting (8.0%) was the main type of malnutrition in 5-19 years with a prevalence of malnutrition of 19.6% (27/138; 95% CI: 13.8-26.9%) in this group age. Prevalence of malnutrition in this age group was significantly higher in female compared to male (24.7% vs. 10.2%, *p* = 0.0001). One participant had severe stunting while no severe underweight case was reported.

In those aged ≥ 20 years, the prevalence of malnutrition was 77.1% (84/109; 95% CI: 68.3-84.0%) and was mainly represented by overweight (33.0%) and obesity grade I (25.7%). The same pattern was observed in male and females.

### 4.5. Proportion of Different Types of Malnutrition by Malaria Status

**Figure 3:**
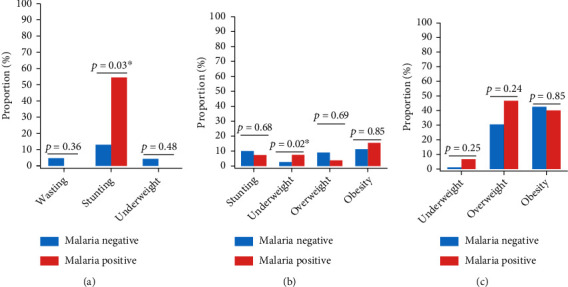
Proportion of the different types of malnutrition with respect to malaria status in participants aged 0-5 years (a), 5-19 years (b), and 20+ years (c). Fisher's exact test was used to make comparisons, ^∗^Statistically significant at *p* value < 0.05.

In the 0-5-year-old group, the proportion of stunting was significantly higher in malaria-infected children compared to their uninfected counterparts (54.54% vs. 13.04%, *p* = 0.03) (Figure 3(a)). In those aged 5-19 years, the proportion of underweight was about three times higher in malaria infected (7.41% vs. 2.70%, *p* = 0.02) (Figure 3(b)). In adults, no statistically significant difference was found between the two malaria groups for underweight, overweight, and obesity (Figure 3(c)).

### 4.6. Factors Associated with Undernutrition among <5 Years and 5-20 Years

The risk of being stunted was fivefold (_A_RR = 4.70; 95% CI: 1.34-7.09; *p* = 0.02) higher in malaria-infected individuals as compared to uninfected ones. No association was found with gender (_A_RR = 1.33; 95% CI: 0.30-2.75; *p* = 0.76), age (_A_RR = 0.69; 95% CI: 0.25-19.5; *p* = 0.49), and study site (_A_RR = 0.41; 95% CI: 0.08-1.54; *p* = 0.23).

In those aged 5-20 years, no statistically significant association was found between gender, study site, and different types of undernutrition (*p* < 0.05). Likewise, the association between malaria and forms of undernutrition was not significant although the risk for stunting (_A_RR = 1.80; 95% CI: 0.16-2.99; *p* = 0.78) and underweight (_A_RR = 1.97; 95% CI: 0.29-7.28; *p* = 0.78) was higher in people infected with malaria parasites. Gender and study sites were not associated with the risk for stunting and underweight among this age group.

### 4.7. Prevalence of Malaria and Malnutrition Cooccurrence and Associated Factors

The overall prevalence of malaria and malnutrition was 8.5% (24/281; 95% CI: 5.8-12.4%). In addition, 34.5% (97/281; 95% CI: 29.2-34.5%) and 10.3% (29/281; 95% CI: 7.3-14.4%) of the study population were suffering from either malnutrition or malaria only, respectively. The rest of persons were exempted from malaria and malnutrition.

**Figure 4:**
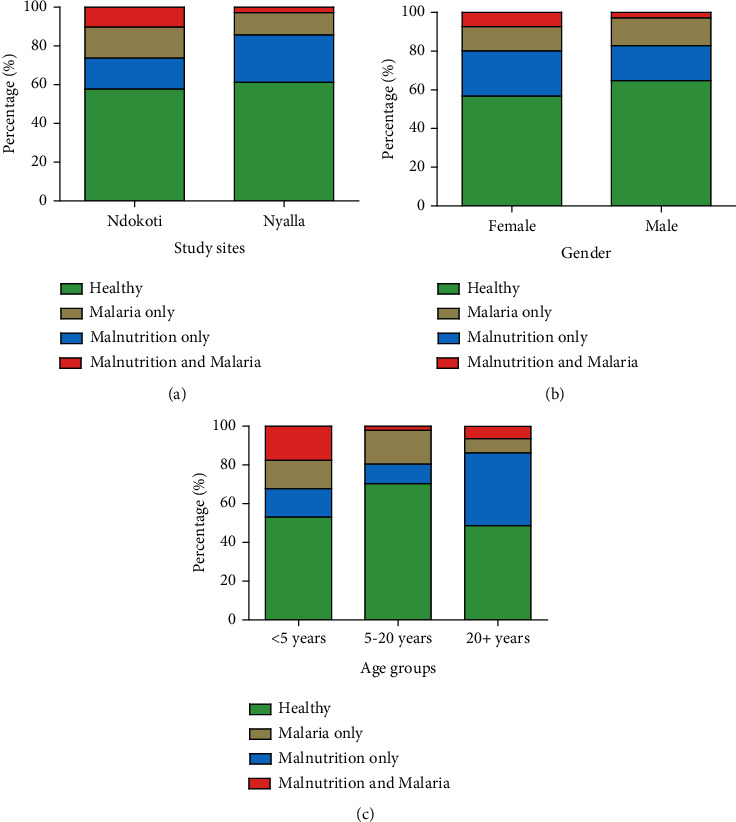
Malaria and nutritional status by study site (a), gender (b), and age (c).

As presented in Figure 4, rates of individuals presenting both malaria and malnutrition were significantly higher in those living in Ndokoti (*χ*^2^ = 8.456; df = 3; _A_RR = 0.39; 95% CI: 0.15-0.99; *p* = 0.04) and children under five (*χ*^2^ = 95.343; df = 6; *p* < 0.0001) compared to their counterparts living in Nyalla and those aged 5-19 years (_A_RR = 0.13; 95% CI: 0.03-0.52; *p* = 0.003). The prevalence of malaria and malnutrition was similar between females and males (*χ*^2^ = 0.754; df = 3; _A_RR = 0.68; 95% CI: 0.25-1.88; *p* = 0.86).

### 4.8. Influence of Malaria Infection and Gender on Anthropometric Index

The influence of malaria and gender on nutritional index is presented in Table 5. Values of WAZ were significantly lower in individuals infected with malaria parasites compared to their uninfected counterparts (1.23 ± 1.42 vs. 0.32 ± 0.22; *p* = 0.003). Likewise, the mean BMI value was significantly lower in malaria-infected individuals (21.28 ± 6.63 vs. 23.72 ± 6.96; *p* = 0.02). Besides, the same pattern was observed only among females for these both anthropometric indexes while among males, no statistically significant difference was found (*p* > 0.05).

### 4.9. Type of Malaria and Anthropometric Indexes

The variation of anthropometric indexes with respect to the type of malaria is summarized in Table 6. No statistically significant difference was found between the different clinical groups (i.e., asymptomatic malaria, symptomatic malaria, and no malaria).

## 5. Discussion

This study focuses on the association between malaria and malnutrition in Douala, Cameroon. Females accounted for most of the participants, and this is consistent with national demographic data [25, 26]. The prevalence of malaria infection was 18.9% in the study population. This finding is lower than 45.47% and 27.54% reported in the same town by Lehman et al. [11] and Mogtomo et al. [27], respectively. Difference in study population, study period, sample size, and malaria diagnosis method could explain this variation. For instance, Lehman et al. assessed the malaria prevalence among schoolchildren even though they used LED microscopy for malaria diagnosis as in our study [11].

Asymptomatic malaria accounted for 84.9% of all malaria cases, and this is consistent with previous studies conducted in the same town [11, 27–29], and other regions of the country [30, 31]. This means that participants would have acquire an immunity which prevents them from developing signs/symptoms of the disease but does not protect them from the infection by the parasite [32]. We previously outlined the importance of asymptomatic carriers of malaria parasites in maintaining malaria transmission as parasite reservoirs and the need for their active detection and treatment for more effective malaria control [11].

Malaria prevalence significantly varied with study site. This is consistent with many reports which outlined the spatial variability of malaria risk and the importance of environmental factors on the distribution of the disease [11, 33]. Risk and prevalence of malaria infection were significantly lower among ≥20 years as compared to less than 5 years. This is in line with previous findings in the same town [11]. This can be due to immune system which is weaker in less than five years in malaria endemic areas. In general, acquired immunity to malaria is lower in children as compared older individuals, and this is particularly seen in high malaria endemic areas [34, 35]. In such areas, the immune system becomes increasingly strong following repeated infectious bites of vector *Anopheles* mosquitoes. This immunity protects individuals from nonsevere and severe malaria [34–36]. Some previous studies reported a high malaria transmission level in the Douala town, where the present study was conducted [37, 38]. Thus, it is likely that host immunity played a key role in the risk of malaria among participants.

The prevalence of malnutrition was 43.1% in this study. This high burden of malnutrition was also reported in other studies conducted in Cameroon [9, 10, 39–43]. Nutrition plays an essential role in building up and maintaining health; and malnutrition appears to elicit susceptibility to a wide range of diseases. Overall, no association between malnutrition and age was found, and this is consistent with findings by Nkuo-Akenji et al. [39] and Gone et al. [8] in Cameroon and Ethiopia, respectively. However, some previous studies in Cameroon present a different picture [9, 10, 42, 43].

The prevalence of undernutrition was higher among less five years, and this is consistent with findings from several studies that reported highest rates of malnutrition in their youngest age categories [9, 10, 43]. These authors investigated malnutrition in 6-17 years (Adamawa Region, Cameroon) and children under five (Littoral Region, Cameroon) and reported highest malnutrition rates in <15 years and 0-6 months, respectively. This can explain why those under five years old were at higher risk of malaria as to the prevalence of undernutrition is high in this group, with subsequent weaker immunity [14, 42]. Undernutrition may be due to the lack of adequate complementary foods, both in nutrient content and in amount of intake to supplement breast milk given the first 2 years of life is very important for optimal growth of children under five [8, 39]. This is supported by some authors who associated the nutritional problems found in those under five years old (0-24 months) in the Bangang rural community (West Cameroon) to low knowledge of feeding practices by mothers [40, 41]. To be noted, the risk for gastrointestinal pathogens carriage, another additional cause of malnutrition, increases with the introduction of complementary foods in this age group [10, 44].

Stunting was the commonest type of undernutrition among <5 years and 5-19 years. This is in line with works by Sumbele et al. in the South West region [10, 45], Mananga et al., Nolla et al., and Dapi Nzefa et al. in the West region [40–42], and Ida Penda et al. [43] and Okalla Ebongue et al. [46] in the Littoral region who estimated prevalence at 17.1%, 23.7%, 42.2%, 41.26%, 16.4%, 63.6%, and 18.2%, respectively. Stunting is known as an excellent surrogate index of chronic malnutrition while wasting is a reflection of severe and acute process, and underweight a complex mixture of many factors such as poor nutrition/eating habits, chronic illness, or even substance abuse [10, 47].

Obesity and overweight were mainly diagnosed in participants aged > 20 years. Our result is higher than the 4.1% found by Mandengue et al., but consistent with reports by Epacka Ewane et al. and Koanga et al. [48–50]. Obesity is an emerging public health concern for African and Asian countries owing to change in food habits and lifestyle (overeating, low energy expenditure, and physical inactivity) [51, 52]. In this latest report, the Organization for Economic Cooperation and Development outlined that The United States of America, Mexico, and New Zealand are the countries where the obesity prevalence in adults were highest [52, 53]. In sSA countries, obesity prevalence has gradually increased this four last decades, especially in countries in the northern (Libya, Egypt), southern (South Africa, Zimbabwe, Namibia, Botswana), and central (Cameroon, Gabon) parts of the continent [52]. The consequences of obesity and overweight on health are enormous, ranging from increased death risk to the appearance of many disorders including metabolic syndrome and diabetes that weakens the quality of life and standard of living of individuals [48].

The risk of stunting was higher in malaria infected among children under five. Previous studies reported similar findings, including a case-control study conducted among children of the Adami Tulu district, Ethiopia [54]. Conversely, our finding disagrees with that of Oldenburg et al. in Nigerian children under five [7]. In addition, malaria infection has negatively affected weight-for-age *Z* score (WAS) and BMI, as mean values of these parameters were lower in malaria-infected individuals. Malaria is known to be associated with poor nutritional outcomes [8, 54], especially with stunting where the disease may predispose to acute weight loss in children [55].

The prevalence of participants with malnutrition and malaria was 8.5%, and this was higher in females compared to males. A meta-analysis study outlined a higher risk of low birth weight in pregnant women with malaria and malnutrition compared to those with only one of these diseases or none [56]. This finding outlined the long-term deleterious effects of both diseases on human health and the need for more attention, especially in our setting where such studies are missing.

This study has several limitations. First, a low number of wasting and underweight precluded the ability to assess the implication of malaria as a risk factor. Second, the presence of unmeasured confounders could have explained certain association found in this study. The nature of the study (cross-sectional study) did not allow for elucidating the temporality between malnutrition and malaria. However, this study is first to address the association between malaria and malnutrition in the town of Douala, the economic capital city of Cameroon. It documents the burden of malnutrition and malaria in our setting in a context of achieving the sustainable development goals (SDGs). Details on food habits and physical activity of participants were not captured, thereby limiting more detailed analyses of the findings found in this study. A low number of malaria-infected participants were symptomatic. In this context, we were unable to do comparisons of the proportion of different types of malnutrition (stunting, wasting, underweight, overweight, and obesity) between asymptomatic and symptomatic malaria cases. LED-based FM was used for malaria diagnosis. This technique does not allow for malaria species diagnosis and quantitative assessment of parasitaemia, which are major drawbacks of this technique. Parasitaemia is commonly determined to assess malaria severity [57]. In addition, asymptomatic and submicroscopic parasitaemia influence nutritional status-assessing parameters such as haemoglobin [58, 59]. However, this technique can be comfortably used in our context since species discrimination is not a problem as *Plasmodium falciparum* is involved in >90% of malaria cases [60, 61]. In addition, LED-based FM has been shown to be as performant as the light microscopy considered as the reference method. The authors outlined that LED-based FM had better operational characteristics than light microscopy [18].

## 6. Conclusion

The present study showed that malaria and malnutrition are prevalent in Douala. It also pointed out the deleterious effects of malaria on nutritional status and vice versa, as well as the influence of host (gender and age) and environment factors on the association between both diseases. Early detection and treatment of these ailments would reduce morbidity and mortality due to malaria and malnutrition in Cameroon. There is also a need for more studies on the topic in the 10 regions of the country to have a clear picture of the burden due to these two public health concerns. Finally, the national malaria control programme should integrate the control of malnutrition in its guidelines in order to efficiently manage malaria in the country. The implementation and scale-up of these different strategies could achieve WHO malaria control and elimination goals, along with mitigating deleterious effects of malnutrition.

## Figures and Tables

**Table 1:** Distribution of study population.

Variables	Ndokoti (*n* = 106)	Nyalla (*n* = 175)	Total (*N* = 281)	*p*
Gender				
Female	73 (68.9)	103 (58.9)	176 (62.6)	0.12
Male	33 (31.1)	72 (41.1)	105 (37.4)	
Age (years)				
<5	12 (11.3)	22 (12.6)	34 (12.1)	0.76
5-19	50 (47.2)	88 (50.3)	138 (49.1)	
≥20	44 (41.5)	65 (37.1)	109 (38.9)	

Data are presented as frequency (percentage). Pearson's chi-square test was used to compare percentage. Statistically significance was set at *p* < 0.05.

**Table 2:** Malaria prevalence by study site, gender, age, and nutritional status.

Variables	*N*	Malaria infected *n* (%)	_R_RR (95% CI)	*p* ^a^	_A_RR (95% CI)	*p* ^b^
*Study sites*						
Ndokoti	106	28 (26.4%)	Reference		Reference	
Nyalla	175	25 (14.3%)	0.53 (0.31-0.89)	0.01^∗^	0.59 (0.33-0.99)	0.04^∗^
*Gender*						
Female	176	35 (19.9%)	Reference		Reference	
Male	105	18 (17.1%)	0.86 (0.50-1.40)	0.57	0.82 (0.46-1.34)	0.47
Age (years)						
<5	34	11 (32.4%)	Reference		Reference	
5-19	138	27 (19.6%)	0.61 (0.29-1.11)	0.11	0.63 (0.29-1.18)	0.16
≥20	109	15 (13.8%)	0.42 (0.19-0.87)	0.02^∗^	0.48 (0.17-0.97)	0.01^∗^
BMI (kg/m^2^)	247	—	0.94 (0.90-0.99)	0.02^∗^	0.95 (0.88-1.02)	0.16
Nutritional status						
Nourished	160	29 (18.1%)	Reference		Reference	
Malnourished	121	24 (19.8%)	1.10 (0.69-1.72)	0.72	1.68 (0.95-2.63)	0.07

^a^Univariate logistic regression was used. ^b^Multivariate logistic regression was used. _R_RR: raw risk ratio. _A_RR: adjusted risk ratio. 95% CI: confidence interval at 95%. BMI: body mass index. NA: not available ^∗^Statistically significant at *p* < 0.05.

**Table 3:** Factors associated with malnutrition.

Categories	*N*	Malnourished *n* (%)	_R_RR (95% CI)	*p* ^a^	_A_RR (95% CI)	*p* ^b^
Study sites						
Ndokoti	106	46 (43.4%)	Reference		Reference	
Nyalla	175	75 (42.9%)	0.99 (0.73-1.27)	0.93	1.11 (0.77-1.45)	0.54
Gender						
Female	176	77 (43.8%)	Reference		Reference	
Male	105	44 (41.9%)	0.96 (0.70-1.23)	0.76	0.97 (0.66-1.31)	0.85
Age (years)						
<5	34	10 (29.4%)	Reference		Reference	
5-19	138	27 (19.6%)	0.66 (0.32-1.24)	0.21	0.70 (0.33-1.30)	0.28
≥20	109	84 (77.1%)	2.62 (1.99-3.02)	<0.0001^∗^	2.70 (2.08-3.07)	<0.0001^∗^
Malaria						
Negative	53	24 (45.3%)	Reference		Reference	
Positive	228	97 (42.5%)	1.06 (0.74-1.39)	0.72	1.31 (0.91-1.66)	0.13

^a^Univariate logistic regression was used. ^b^Multivariate logistic regression was used. _R_RR: raw risk ratio. _A_RR: adjusted risk ratio. 95% CI: confidence interval at 95%. ^∗^ Statistically significant at *p* < 0.05.

**Table 4:** Types of malnutrition by age groups and gender.

Age groups	Female		Male		Total	
	*n*	%	*n*	%	*N*	%
<5 years (*n* = 34)						
Stunted	5	29.4	3	17.6	8	23.6
Wasting	0	0.0	1	5.9	1	2.9
Stunted + underweight	0	0.0	1	5.9	1	2.9
Total	5	29.4^∗^	5	29.4^#^	10	29.4^†^
5-19 years (*n* = 138)						
Stunted	8	9.0	3	6.0	11	8.0
Overweight	7	7.9	1	2.1	8	5.8
Underweight	4	4.5	0	0.0	4	2.9
Obesity	1	1.1	1	2.1	2	1.4
Obesity + stunted	1	1.1	0	0.0	1	0.7
Stunted + underweight	1	1.1	0	0.0	1	0.7
Total	22	24.7^∗∗^	5	10.2^##^	27	19.6^††^
≥20 years (*n* = 109)						
Overweight	19	27.1	17	43.6	36	33.1
Obesity I	17	24.3	11	28.2	28	25.7
Obesity II	11	15.7	2	5.3	13	11.9
Obesity III	5	7.1	0	0.0	5	4.6
Underweight	2	2.9	0	0.0	2	1.8
Total	54	77.1^∗∗∗^	30	77.1^###^	84	77.1^†††^

Percentage is computed using the total number of each age category and gender as denominator (*N* = 17^∗^, *N* = 89^∗∗^, and *N* = 70^∗∗∗^ for the second column, *N* = 17^#^, *N* = 49^##^, and *N* = 39^###^ for the third column, and *N* = 34^†^, *N* = 138^††^, and *N* = 109^†††^ for the fourth column).

**Table 5:** Effect of malaria infection, age group, and gender on anthropometric parameters.

Parameters	Females			Males			All		
	Uninfected	Infected	*p* ^e^	Uninfected	Infected	*p* ^e^	Uninfected	Infected	*p* ^e^
WHZ^a^	2.87 ± 2.68	1.88 ± 2.82	0.43	1.83 ± 2.13	2.03 ± 0.13	0.85	2.27 ± 2.38	1.93 ± 2.31	0.67
WAZ^a^	1.58 ± 0.25	0.28 ± 0.25	0.001^∗^	0.87 ± 0.23	0.92 ± 0.44	0.33	1.23 ± 1.42	0.32 ± 0.22	0.003^∗^
HAZ^b^	0.19 ± 0.31	−0.81 ± 0.43	0.10	0.75 ± 0.97	−0.69 ± 0.42	0.49	0.41 ± 0.43	−0.77 ± 0.32	0.15
BMIAZ^c^	0.84 ± 0.24	0.91 ± 0.43	0.88	1.12 ± 1.76	0.98 ± 1.33	0.79	0.96 ± 0.17	0.94 ± 0.32	0.95
BMI (kg/m^2^)^d^	24.52 ± 7.54	21.13 ± 7.14	0.01^∗^	22.44 ± 5.72	21.57 ± 5.69	0.56	23.72 ± 6.96	21.28 ± 6.63	0.021^∗^

Data are presented as mean ± standard deviation (SD), WHZ: weight-for-height *Z* score, WAZ: weight-for-age *Z* score, HAZ: height-for-age *Z* score, BMI: body mass index, BMIAZ: BMI-for-age *Z* score. ^a^Computed for participants aged 0-5 years old. ^b^Computed for participants aged 0-5 years old and 5-19 years old. ^c^Computed for participants aged 5-19 years old. ^d^Computed for participants aged 20 years old and above. ^e^Student's *t*-test or Mann-Whitney's tests were used to compare mean values. ^∗^Statistically significant at *p* < 0.05.

**Table 6:** Variation of anthropometric indexes with regard to type of malaria.

Parameters	Clinical groups			
	Asymptomatic malaria (*n* = 44)	Symptomatic malaria (*n* = 9)	No malaria (*n* = 301)	*p* ^e^
WHZ^a^	2.24 ± 0.79	1.93 ± 0.00	2.27 ± 2.38	0.98
WAZ^a^	0.29 ± 1.23	1.26 ± 0.00	1.23 ± 1.42	0.15
HAZ^b^	1.90 ± 3.075	0.33 ± 0.00	0.41 ± 0.43	0.09
BMIAZ^c^	0.22 ± 1.31	1.38 ± 0.54	0.96 ± 0.17	0.11
BMI (kg/m^2^)^d^	28.29 ± 4.84	30.70 ± 7.91	23.72 ± 6.96	0.92

Data are presented as mean ± standard deviation (SD), WHZ: weight-for-height *Z* score, WAZ: weight-for-age *Z* score, HAZ: height-for-age *Z* score, BMI: body mass index, BMIAZ: BMI-for-age *Z* score. ^a^Computed for participants aged 0-5 years old. ^b^Computed for participants aged 0-5 years old and 5-19 years old. ^c^Computed for participants aged 5-19 years old. ^d^Computed for participants aged 20 years old and above. ^e^The Kruskal-Wallis or analysis of variance (ANOVA) tests were used to compare mean values. Statistical significance was set at *p* < 0.05.

## Data Availability

All data generated or analysed during this study are included in this paper.

## Abbreviations

95% CI: Confidence interval at 95%
_A_RR: Adjusted risk ratio
ANOVA: Analysis of variance
BMI: Body mass index
BMIAZ: Body mass index for age *Z* score
FM: Fluorescence microscopy
HAZ: Height-for-age *Z* score
LED: Light-emitting diode
NA: Not available
OR: Odds ratio
_R_RR: Raw risk ratio
SD: Standard deviation
sSA: Sub-Saharan Africa
WAZ: Weight-for-age *Z* score
WHO: World Health Organization
WHZ: Weight-for height *Z* score.
